# Innocent Laryngeal Growths—II

**Published:** 1909-01-09

**Authors:** 


### Laryngology and Rhinology.

### INNOCENT LARYNGEAL GROWTHS—II.

The diagnosis of the existence of a laryngeal tumour is simply a matter of inspection of the larynx with the mirror, and the question is only mentioned because growths not infrequently occur at the anterior part of the glottis, where they may easily escape observation if the epiglottis be dependent. It is obviously necessary to examine every part of the larynx before excluding the presence of a tumour. On making the patient sing a high note or rapidly ascend the scale the epiglottis is generally so tilted forwards as to allow a good view of the anterior commissure, and sometimes the effect is obtained during the sharp inspiration which follows a prolonged phonation; more rarely it is necessary to employ an epiglottis retractor, after thorough application of cocaine. In very young children inspection of the larynx is difficult, but it can nearly always be accomplished if the little patient be wrapped in a towel and firmly held on the knee of an assistant; in these cases an epiglottis retractor is most useful to control the tongue. A general anæsthetic may be necessary in the case of older children who are very intractable. A rapid glance is sufficient to establish the diagnosis. Hoarseness of long duration, occurring in a child free from severe constitutional disease, is nearly always due to papilloma.

It is not always possible to make a differential diagnosis between the various forms of innocent tumours, nor indeed is this of practical importance. The important thing is to distinguish these innocent growths from the granulomata on the one hand, and from the malignant neoplasms on the other. Only in their most exceptional manifestations do tuberculous and syphilitic laryngitis in any way resemble innocent tumours; in both these affections sprouting granulations are often seen, but nearly always in association with definite infiltration or ulceration, and solitary innocent tumours occur very rarely in the posterior commissure or on the posterior third of the vocal cord, where tuberculous outgrowths are common. Nevertheless, in rare cases tuberculomata are found in the absence of all the ordinary appearances of tuberculous laryngitis, and closely resemble simple papillomata; it is said that they usually occur when the pulmonary disease is in a very early stage, and are thereby rendered doubly difficult of diagnosis.

The diagnosis from epithelioma is of the greatest importance; papilloma may occur in old men, and epithelioma in young ones. The latter may first appear as a single warty outgrowth; a single growth appearing at a level posterior to the centre of the vocal cord should always excite suspicion. An epithelioma infiltrates the substance of the cord from the first, and on this the most characteristic signs depend, even though the actual infiltration may be invisible. Thus an innocent tumour is pedunculated, and; though it may overlie its pedicle, it is seen on phonation to be freely movable. An epithelioma is always more or less sessile, and is accompanied from the outset by a limitation or sluggishness of the movement of the cord; this sign, pointed out by Semon, is of great importance in the diagnosis of malignancy. Some thickening of the tissues, also, may often be seen beyond the obvious limits of an epithelioma. It need hardly be said that many epitheliomata originate in a form totally unlike that of any innocent tumour.

An innocent growth, so situated as not to interfere with the movements of the cords, may produce no symptoms whatever. The chief symptom is hoarseness, and a peculiar gruff character of the voice is highly characteristic. Some discomfort on swallowing may be caused by a tumour at the upper aperture. Dyspnœa is due to mechanical blocking of the lumen, and may be extraordinarily slight, even in cases of a very large tumour, if it has grown slowly. This symptom is most common in children with multiple papillomata, and is then generally paroxysmal in character, being complicated by spasm of the glottis.

Such innocent tumours as cause no symptoms may very well be left alone. Small singer's nodules and inflammatory growths at the seat of election diminish, and often disappear, under treatment based on rest of the larynx and avoidance of irritation; and after instrumental removal of a growth such treatment is most important in order to avoid resurrence. If there be any inflammation or thickening of the cords, especially if the patient be a professional voice-user, absolute silence is indicated for two or three weeks after the operation, and the question of correct voice-production must be thoroughly considered. An oily solution, such as paraffinum liquidum containing 1 per cent. of menthol, should be used in an atomiser, dusty atmospheres must be avoided, and attention given to the condition of the nose and pharynx and to the general health. The stump of a papilloma may be touched with the galvano-cautery, and this is also a valuable method of treating minute nodules. Various caustics have been recommended for recurrences and for small growths; perhaps the best is an alcoholic solution of salicylic acid up to 6 or 8 per cent.

But complete removal with instruments is the proper method of treatment in the large majority of cases. The safe performance of this delicate operation demands considerable dexterity and technique, and need not be thoroughly discussed here. The ordinary method is to remove the growth with local anæsethesia under inspection in the laryngoscope; it should be cut rather than torn off, and the forceps should be fashioned accordingly. It is most important that any attempt at removal be made only when the growth is fully and clearly in view. Difficult cases may require training at several sittings, and the patient should always be told that it may not be possible to remove the growth on the first occasion. Multiple papillomata in children are cases of great difficulty. Until lately decidedly the best method was to train the child in intralaryngeal manipulation; this is extremely tedious, but is generally possible in patients over about seven years of age, and when once accomplished the removal of recurrent growths becomes easy—a point of importance, as recurrence is very common. The alternative method has been to operate under chloroform; deep anæsthesia must be induced in the sitting posture, and cocaine must also be applied, so that the method is not without danger. If there is dyspnœa, tracheotomy must be performed before any attempt at removal. The latter operation has also been recommended in the hope that it will induce the growths to disappear; this it rarely does, though they tend to vanish at puberty. The opening in the trachea enables one to wait until the patient's age allows of easier removal, at the risk of pulmonary disease entailed by long wearing of the tube. The recent improvements in methods of direct laryngoscopy, however, have altered the position, and enabled us to deal with these cases with safety and with comparative ease.

